# Recent advancements in the role of phytochemicals and medicinal plants in prophylaxis and management of Alzheimer’s disease

**DOI:** 10.22038/ijbms.2024.77760.16826

**Published:** 2024

**Authors:** Akanksha Mishra, Sairam Krishnamurthy

**Affiliations:** 1 Department of Pharmacology, Institute of Pharmaceutical Sciences, University of Lucknow, Lucknow-226031, U.P., India; 2 Neurotherapeutics Laboratory, Department of Pharmaceutical Engineering & Technology, Indian Institute of Technology (Banaras Hindu University), Varanasi-221005, U.P., India

**Keywords:** Alzheimer’s disease, Dietary supplements, Medicinal plants, Neurodegenerative- disorders, Phytochemicals

## Abstract

Medicinal plants and phytochemicals are some of the major sources in the treatment of various neurodegenerative disorders including Alzheimer’s disease (AD). There is no FDA-approved drug to target AD pathology directly. Full cognitive restoration and management of psychosis-like symptoms are still to be achieved. Being comparatively safer with fewer side effects, medicinal plants have been among the major areas of interest to be researched. Several mechanistic pathways are involved in AD including anticholinesterase activity, glutamate toxicity, free radicals generation, Amyloid β (Aβ) toxicity, inflammation, and mitochondrial dysfunction. Various phytochemicals such as paenol, andrographolide, isoquercitrin, flavonoids, and saponins obtained from different plant sources, various medicinal plants like *Spirulina maxima, Salicornia europaea, Curcuma longa, Citrus Junos Tanaka, Cassiae semen, Centella asiatica* as well as various traditional medicinal plants of China, Asia, Europe, Turkey, and Iran have been found effective against one or more of these targets. Large numbers of clinical trials are under process to evaluate the role of different phytoconstituents in AD management. Out of 143 agents under clinical trials, 119 have been categorized as disease-modifying agents. The present review extensively covers the recent advancements in the usage of phytochemicals and medicinal plants in various experimental AD models. It involves clinical trials and other research works divided into three sections, including those performed *in vitro, in vivo*, and in humans mainly from the last five years along with disease markers and mechanistic pathways involved. However, phytochemicals should be explored further in order to achieve neurorestoration in AD.

## Introduction

**Figure 1:**
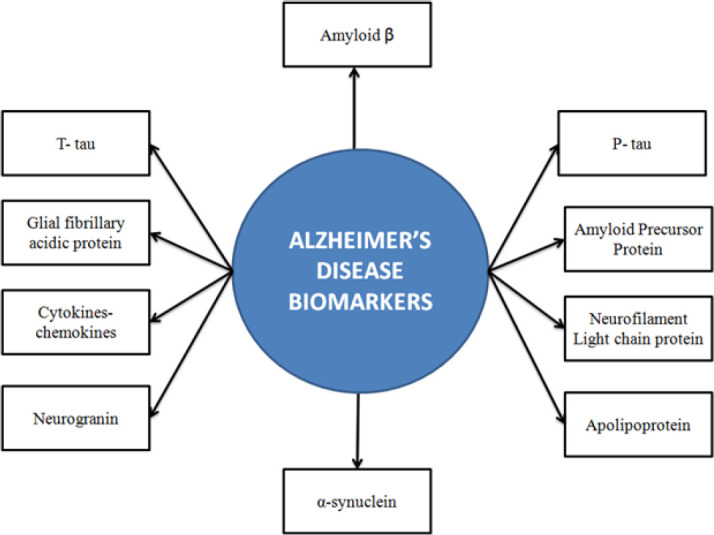
Overview of biomarkers in Alzheimer’s disease (AD)

Alzheimer’s disease (AD), the most common neurodegenerative disorder, is characterized by deficits in cognition in the aging population (1). AD may also contribute to 70% of cases of dementia, a syndrome related to cognitive deterioration beyond the normal consequences of aging. Six and a half million people in the US were suffering from Alzheimer’s dementia in 2022, out of which 73% were of 75 years or older age (2). AD is predicted to affect more than 100 million people worldwide by 2050 (3). As life expectancy is increased globally, it indicates a major economic and societal challenge to patients and their families. The primary symptom of AD includes short-term memory loss, followed by disorientation, mood swings, and behavioral deficits at later stages. During advanced stages, AD leads to loss of body function followed by death (4). Some of the AD biomarkers include amyloid precursor protein (APP), amyloid β (Aβ) peptides, Aβ oligomers, Tau protein, phosphorylated tau (p-tau), neurofilament light chain protein (NfL), Glial fibrillary acidic protein (GFAP), cytokines-chemokines, α-synuclein, apolipoprotein, and neurotrophic factors. Figure 1 shows some of the important AD biomarkers (1, 5).

AD patients are found to be deficient in acetylcholine, a neurotransmitter required for learning and memory. The cholinergic hypothesis of AD refers to the deficiency of acetylcholine, cholinergic neurons, and reduced acetylcholinesterase (AChE) activity (6). AD treatment approaches can be broadly divided into three categories: symptomatic, disease-modifying, and regenerative. At present, there is a lack of Food and drug administration (FDA) approved therapeutic agents that can target AD pathology directly. Current therapeutic approaches consist of symptomatic treatment including glutamate antagonists for antagonism of N-methyl-D-aspartate receptor and cholinesterase inhibitors for agonism for cholinergic system which covers cognition to some extent (4). The complete restoration of cognitive function and management of other symptoms like psychosis and sleep disruption is still to be achieved using current treatment approaches (7).

Apart from synthetic drugs, various bioactive molecules obtained from plants have been traditionally used for various diseases due to high patient compliance and lower side effects. Some commonly used AChE inhibitors, such as rivastigmine and galantamine, are obtained from plants (8). Till now, a number of natural products have been approved by the FDA for the treatment of AD which include but are not limited to huperzine A, a traditional Chinese medicine; galantamine, an alkaloid present in Amaryllidaceae family; sodium oligomannate, derived from marine brown algae and approved conditionally in China (9); and rivastigmine, a semi-synthetic derivative of physostigmine (isolated from calabar bean)(10). Many phytoconstituents and medicinal plants are being extensively studied in order to develop effective therapeutic agents for the management of AD including polyphenols (flavonoids)(11), garlic extracts (12), andrographolide (ANDRO) from *Andrographis paniculata *(13), a pyrroloquinazoline alkaloid vasicinone from *Adhatoda vasica *(14), *Asparagus racemosus *(15), paenol from* Paeonia suffruticosa *(16), Mulberry Diels-Alder-type adducts (MDAAs) from *Morus alba *(17), Genistein from Soybean extracts (18), Trans-Crocin 4 and trans-Crocetin from *Crocus sativus *(19**)**, Safranal from *Crocus sativus *(20),* Spirulina maxima* extract (21), Sesamin and Astaxanthin from sesame (22), *Salicornia europaea* L. extract (23), flavonoids & saponins from *Bacopa floribunda *(24)*, *phytochemicals from *Curcuma longa* and *Citrus Junos Tanaka *(25)*, *phytochemicals from *Withania somnifera *(26),* Clausena harmandiana* root bark extract (27), *Centella asiatica *(28), Jatamansinol from *Nardostachys jatamasi *(29), *Mangifera indica *(30), Silibinin from *Silybum marianum *(31), and Betanin and Glycine betain from *Beta vulgaris *(32).

Many clinical trials are being conducted to evaluate the role of different phytoconstituents in the management of AD. As of January 2022 data, 143 agents are under clinical trials for the management of AD, out of which 119 agents are classified for disease modification (33). The current review covers the results and details of some of the recent clinical trials and other studies mainly from the last five years (4 studies reported in the year 2024, 2 in 2023, 25 in 2022, 9 in 2021, 9 in 2020, 6 in 2019, 3 in 2018, 1 ongoing, and other clinical trials) for the usage of various plant sources and their phytochemicals in AD. The studies included are divided into three categories to observe the effects of multiple phytochemicals and plant extracts on different AD markers investigated in *in vivo* and *in vitro* cell cultures and humans. The present review will help the researchers get extensive information about the current treatment approaches under clinical or preclinical studies.

**Phytochemicals in AD**

***In vivo studies***

*Paenol from Paeonia suffruticosa*

Paeonol, a phenolic compound present in Cortex Moutan (a root bark of *P. suffruticosa* Andr.) and a traditional Chinese medicine at 100 mg/kg was observed to be effective against Streptozotocin (STZ) induced murine model of sporadic AD when administered orally from day 0 to day 24 post-STZ. The effect was dose-dependent, as a 25 mg/kg dose was found to be ineffective. Being a low molecular weight compound, it easily crosses the blood-brain barrier. STZ causes impairment in brain glucose metabolism, which is directly related to the quantity and physiology of remaining neurons. Apart from the anti-oxidant and anti-inflammatory effects, paenol has hypoglycemic activities due to which it decreases glucose hypometabolism in the brain in STZ-induced AD rats (16).

*Petroselium crispum extract*

*P. crispum *(commonly known as Parsley, family *Apiaceae*) is a natural source of minerals and vitamins and reported to be effective against diabetes, inflammation, oxidative stress, and apoptosis. *P. crispum *extract (2 g/kg p.o.) was administered for 14 days in the Scopolamine-induced AD model in rats. It showed protection against scopolamine-induced cognitive dysfunction in the Morris water maze and novel object recognition tests. Additionally, it decreased AChE activity and mitochondrial-mediated apoptosis and proved to be an anti-oxidant due to the alleviation of malondialdehyde and increased glutathione (GSH) levels in various brain regions. M1 is a type of muscarinic receptor highly expressed in the brain’s hippocampus and frontal cortex regions. It is involved in cognitive function, and its deficiency may cause memory impairment. The extract also increased M1 receptor expression in hippocampus and frontal cortex regions in scopolamine-induced AD rats, proving its efficacy against AD (34).

*Brewed coffee*

Coffee, one of the most used beverages worldwide, was recently evaluated for its role against memory impairment in AD. Brewed coffee (BC) was given to male Wistar rats at 5.7 ml/kg (10 g in 100 ml water) for three weeks after the STZ-induced AD model. Excitatory post-synaptic potentials (EPSPs) were observed to be significantly increased by BC in STZ-induced AD rats, indicating memory improvement. Additionally, BC treatment significantly decreased Aβ plaques, a significant AD marker in the hippocampal region of STZ-induced AD rats. Still, more research is required to further clarify the mechanism of BC-induced neuroprotection (35).

*Andrographolide from Andrographis paniculata*

ANDRO, a bioactive ingredient present in *A. paniculata*, is reported to have therapeutic effects in the central nervous system (CNS)-related disorders, including neurodegeneration (36). Recently, the impact of long-term ANDRO treatment was evaluated in rats using the STZ-induced AD model. ANDRO was given to rats as 2 mg/kg IP three times a week for four weeks, initiated one hour post-surgery. Short-term spatial memory in the Y-maze and object-recognition test was improved, astrocyte activation (role in the intro) was increased in the prefrontal cortex, and activated microglia cell count was decreased in the hippocampus by ANDRO in STZ-induced AD rats. Overall, ANDRO was proven to be therapeutic against neuroinflammation and cognitive dysfunction in AD models in rats (37).

*Allium cepa and Allium sativum extracts*

A study has been published to evaluate the mitigation effects of fresh root extracts of *A. cepa* (onion) and *A. sativum* (garlic) against neurodegeneration caused by aluminum chloride (AlCl_3_) in male Sprague-Dawley rats. The roots were evaluated for the presence of phenolic and flavonoid contents, and antibacterial, antifungal, and anti-oxidant activity. 1, 2, and 3 mg/kg of onion and garlic root extracts were administered to different animal groups for 30 days orally after 45 days of AlCl_3_ administration to induce AD. Both the root extracts at 100 and 200 mg/ml concentration showed anti-bacterial activity against *E.coli, S. aureus,* and *P. aeruginosa*. It also showed anti-fungal activities against fungi (*A. niger, P. verrucosum, F. proliferatum, and A. ochraceus*), and anti-oxidant activity in 2,2-diphenyl-1-picrylhydrazyl (DPPH), ABTS [2,2’-azino-bis(3-ethylbenzothiazoline-6-sulfonic acid)] and ferric reducing ability of plasma (FRAP) assay. Both extracts were observed with high content of total phenolic and avonoid content. Different doses of extracts also decreased histopathological lesions, apoptotic genes, DNA damage, and generation of reactive oxygen species (ROS) in the brain tissues of AD rats, indicating its neuroprotective effects. The underlying mechanism may be the anti-oxidant activity and regulation of genetic expression patterns of flavonoids (38).

*Pluchea lanceolata extract*

*P. lanceolata* (source of “Rasna”; Family *Asteraceae*) is a medicinal plant used for the traditional treatment of psoriasis, bronchitis, cough and inflammation, laxative, analgesic nerve tonic, dementia, and other nervous system diseases. Various *P. lanceolata* (PL) chemical constituents include D-glucoside, β-sitosterol, quercetin, isorhamnetin, pluchine, moretenol and pluchiol (39). Root and leaf extracts of PL were administered to Wistar rats orally at 200 and 400 mg/kg doses in AlCl3-induced AD rats. In the elevated plus maze and pole climbing test, 200 and 400 mg/kg doses of PL were observed to be effective, indicating its role in cognitive function, learning, and memory. Antioxidant enzymes were increased in blood serum and brain tissues of PL- PL-administered rats. Synaptic and cognitive functions are regulated by various neurotransmitters. Brain neurotransmitters such as serotonin, dopamine, noradrenaline, and acetylcholine were increased by PL administration in AlCl_3_-induced AD rats. Histology of the cortex and hippocampus showed minimum hemorrhage and neuronal vacuolation in PL-treated AD rats, indicating cellular protection. Therefore AlCl_3_ AlCl3-induced neurotoxicity was reversed by PL treatment dose-dependently in the AD model (40).

Some of the other phytochemicals investigated *in vivo *for the management of AD are shown in Table 1.

***Cell cultures/in vitro***

*Evolvulus alsinoidesextracts*

*E. alsinoides, *commonly recognized as Dasapushpam in Ayurveda is a perennial herb of India. Its major phytoconstituents include umbelliferon, triacontane, betaine, scopoletin, sitosterol, shankpushpine, and scopolin. The plant extracts were earlier used by local practitioners traditionally as a brain tonic and cognition booster to rejuvenate the nervous system and for the treatment of neurodegenerative disorders (41). Recently neuroprotective potential including enzyme-inhibitory activity of its leaf extract has been investigated in AD models *in vitro*. Leaf extracts of *E. alsinoides *showed the presence of phenolics, tannins, and flavonoids. Five different concentrations (100-500 µg) of leaf extract were evaluated for anti-oxidant activity in FRAP and DPPH assays. Seven concentrations (25-500 µg) were evaluated for anticholinesterase activity. IC50 (half maximal inhibitory concentration) values were obtained for DPPH assay (52.43+0.2 µg/ml), FRAP assay (41.58+0.03 µg/ml), and anticholinesterase activity (4.46+0.03 µg/ml). Therefore the obtained enzyme inhibitory and anti-oxidant activities support its traditional use against AD. However, further studies are required to explain its exact role and mechanism in the treatment of AD (42).

*Morin and isoquercitrin*

Flavonols of natural origin are reported to have anti-oxidant properties. It inhibited aggregation of Aβ and destabilized fibrils *in vitro*. Morin and isoquercitrin are reported to have anti-oxidant and cytoprotective properties. Additionally, morin and isoquercitrin showed anti-apoptotic and anti-inflammatory effects, respectively (43, 44). Recently both phytochemicals have been tested for their efficacy against the amyloid toxicity model in MC65 cells along with their *in vitro* activities against AChE activity and fibrillogenesis of Aβ peptide. Morin and isoquercitrin inhibited the aggregation of Aβ, oxidative stress, caspase-3, and caspase-9 activity in cell cultures (44).

*Mulberry Diels-Alder-type adducts (MDAAs) from Morus alba*

MDAAs are the biosynthesis products of intermolecular cycloaddition of dehydroprenylphenol and chalcone. MDAAs are also isolated naturally from the root bark of *Morus alba *(Family- *Moraceae*), traditional Chinese medicine for the treatment of hypertension, diabetes, inflammation, and hypoglycemia (45). Mulberrofuran C, K, and G alongwith isomulberrofuran G are reported to have therapeutic potential against various targets of AD. Due to its good blood-brain barrier permeability (8.7+ 0.3×10^-6^ cm/s), mulberrofuran K was further studied for its mechanism against glutamate-induced toxicity in the HT22 cell model. It was found to increase GSH levels and suppress ROS production (17).

*Genistein from Soybean extracts*

Genestein, present in soybean (China-originated) extract is classified as isoflavone. It is reported to act against fibrosis, oxidative stress, cancer, and inflammation (46). Additionally, it improved learning and memory in various diseases and mitigated astrogliosis in the AD model (47). Recently its mechanism for cell protection was also elucidated against Aβ_25-35 _–induced AD model *in vitro *via Nrf2/ HO-1/PI3K (nuclear factor erythroid 2- related factor-2/ heme oxygenase -1/ phosphoinositide 3-kinase) signaling. Toxicity was induced by Aβ_25-35 _in SH-SY5Y cells (20 µM) for 24 hr, pretreated by genestein (10, 30, or 50 µM) for 90 min. The expression of HO-1, PI3K p85 phosphorylation along with cell viability was measured. Genestein not only increased PI3K p85 phosphorylation and mRNA, but protein amount of HO-1but also enhanced cell survival. However, on using Nrf2 and PI3K p85 inhibitors, HO-1 levels were not increased in Aβ_25-35 _–induced AD model in SH-SY5Y cells. Overall, the results proved the involvement of Nrf2/ HO-1/PI3K signaling in the neuroprotective action of genestein (18).

*Trans-Crocin 4 and trans-Crocetin from Crocus sativus*

These are obtained from *C. sativus*, a traditional medicine that showed its therapeutic action against different pathologies related to cognitive impairment, apoptosis, and neurotoxicity (48). Trans-crocin 4, the principal constituent of crocin, and trans-crocetin, a distinctive C20 apocarotenoid caused a reduction in Aβ_1-40_ aggregation in PC12 cells and human AD monocytes, respectively (49, 50). Both constituents from *C. sativus* were recently evaluated for their activity *in vitro *against cell-culture models of AD using SHSY5Y cells overexpressing APP and PC12 cells overexpressing hyperphosphorylated tau. Trans-crocin 4 (1 mM) and trans-crocetin (10 µM) were used to treat SH-SY5Y-APP cells for 72 hr. The levels of β-secretase, involved in the amyloidogenic pathway, and γ-secretase involved in the formation of Aβ toxic peptides were decreased by trans-crocin 4 and trans-crocetin in SHSY5Y cells expressing APP. Glycogen synthase kinase-3 beta (GSK3 β) and extracellular signal-regulated kinase (ERK) ½ kinases active forms along with tau phosphorylation were decreased by both the phytoconstituents. Overall, both compounds modulated the amyloidogenic pathway and tau misprocessing in cell culture models of AD (19).

*Safranal from Crocus sativus*

Dried stigma of *C. sativus *commonly known as saffron has been reported to possess expectorant, anti-asthmatic, anti-inflammatory, anti-depressant, anti-nociceptive, and memory-enhancing potential. The constituents including crocin, crocetin, safranal, and carotene-like phenolic compounds are responsible for its antioxidant potential. Safranal is an aldehyde responsible for the odor (51). Recently the therapeutic potential of safranal has been reported in AD model *in vitro*. Safranal proved its efficacy against Aβ_(25-35)_- and hydrogen peroxide (H_2_O_2_)- induced cellular toxicity in PC12 cells by affecting mitogen-activated protein kinase (MAPK) and PI3K pathways. Safranal (2.5, 5, 10, 20, and 40 µM) was used to treat PC12 cells for 120 min, followed by exposure of 25 µM Aβ or 150 µM H_2_O_2_ for 48 hr and 24 hr, respectively. Selected concentrations of safranal were not found cytotoxic. Aβ-induced cell death was decreased by 2.5 µM conc of safranal. H_2_O_2_–induced ROS generation was significantly reduced by all the doses of safranal. 2.5 µM conc inhibited Aβ_(25-35)_-induced apoptosis and decreased the levels of apoptotic proteins [Cyt C, survivin, p44/42 MAPK, PI3 Kinase P85, stress-activated protein kinase/ jun amino-terminal kinases (SAPK/JNK), and caspase 3]. This study proved the anti-apoptotic and anti-oxidant potential of safranal for the treatment of AD (20).

Table 2 Shows some other phytochemicals recently investigated in cell cultures for the management of AD.

***Human studies***

*Cedar leaves*

The fragrance of cedar leaves has been reported to be beneficial for health. Recent reports suggested its role in the improvement of cognition. Cedar leaves (20 gms) were cut, mixed with ethanol 20%, and distilled to ethanol 50% with a rotary evaporator. The product is a type of environmental disinfectant with anti-bacterial activity from which spray and room fragrance dispensers were made. The dispensers were kept in residential areas. Patients having AD-type dementia without olfactory impairment were selected for the study. Patients with mild cognitive impairment (MCI) were excluded. Patients receiving cedar fragrance for 8 weeks showed significant improvement in behavioral and cognitive symptoms of dementia. Since the procedure is not invasive, it is comparatively simple and may decrease nursing care (52).

*Spirulina maxima extract*

*S. maxima*, a microalga is considered safe as food due to the presence of nutrients and other bioactive substances (53). Its 70% ethanol extract (SM70EE) has been shown to reduce memory dysfunction in Aβ and scopolamine-induced mice models of memory impairment (54). Recently, a study reported the effects of *S. maxima* extract on cognition in patients of MCI. The study was performed on 80 subjects in a setup of 12 12-week double-blind, randomized, placebo-controlled clinical trials in which patients were administered SM70EE 1 g/day. The patients were inspected at baseline and the 12^th^ week using the Montreal Cognitive Assessment (MoCA) Korean version. The other parameters investigated were BDNF (brain-derived neurotrophic factor) expressions and Aβ levels. No differences were observed in other secondary validation parameters Aβ and BDNF. However, AD symptoms such as visual learning and working memory along with vocabulary were increased without any adverse effects. It was the first clinical trial showing the positive effects of SM70EE on cognition in patients with early AD suffering from MCI (21).

*Sesamin and Astaxanthin from sesame*

Sesame, a lignan present in sesame extract is majorly used in traditional Chinese medicine. It has been reported to show neuroprotective effects and mitigate the damage induced by cerebral induced ischemia (55). Astaxanthin is classified as carotenoid and is present in crab, salmon, shrimp, and microalgae (56). This strong antioxidant is reported to cross the blood-brain-barrier (57) and reduce brain impairment caused by oxidative stress in animals and humans (58-60). In an earlier study, both the phytochemicals sesamin and astaxanthin have been evaluated for their effects on cognition in patients with MCI in a double-blind, randomized, placebo-controlled pilot study. Patients received two capsules daily for 12 weeks. One capsule consisted of astaxanthin (3 mg) from *Haematococcus pluvialis* and sesamin (5 mg) from *Sesamum indicum *(Indian origin) as major components and dispersants and safflower oil as filling agents. Tests for Central Nervous System Vital Signs (CNSVS) Japanese version and AD Assessment– Cog were performed at baseline and after 6 and 12 weeks of study. Both the processing and psychomotor speeds were improved, indicating better cognition associated with comprehension and complex activity-performance in patients with MCI. No adverse effect related to the phytochemical was observed (22).

*Salicornia europaea L. extract*

*S. europaea*, a halophytic plant, has shown significant neuroprotective effects in several studies. Its positive effects have been also shown against the scopolamine-induced amnesia model in mice (61). Acanthoside B, a phytoconstituent obtained from *S. europaea* ameliorated cognitive dysfunction with negligible adverse effects on amnestic models in mice. The major mechanism responsible for its action includes inhibition of AChE activity, inflammatory cytokines, as well as oxidative stress. *S. europaea* also activates signaling pathways involved in the pathology of AD including neurotrophic tropomyosin receptor kinase B (TrkB) / cAMP response element binding (CREB)/ BDNF (62). Recently a study has reported the positive effects of desalted *S. europaea* L. ethanolic extract (Phytomeal; PM-EE) on 63 patients with memory dysfunction without dementia, who had Korean Mini-Mental State Examination [K-MMSE] score ≥ 23. This was a placebo-controlled, randomized, double-blind 12-week clinical trial. PM-EE was given as a 600 mg/day dose. AD assessment scale – Korean version (ADAS-K) was considered as the primary outcome for the assessment of cognition. After 12 weeks, neither any improvement was observed in the scores of primary outcome, nor on the adverse events. However, while performing a subgroup analysis of 30 subjects suffering from MCI and K-MMSE score ≤ 28, positive effects were observed in the Korean color-word Stroop test for the color-reading score. Adverse events like tinnitus, dyspepsia, and gastroesophageal reflux disease were found to be mild in nature and not related to drug usage. The results indicate that *S. europaea *is not effective for cognition in selected subjects having memory dysfunction without dementia, but it may have positive effects on the frontal executive function in patients suffering from MCI. Therefore further studies are required for a larger duration (23).

*Guilingji capsules*

Guilingji capsules (GLJC), a patented herbal medicine of Chinese origin have been traditionally used in the management of memory dysfunction (63). GLJC is made up of 28 herbs, including *Rehmannia glutinosa* Libosch, *Psoralea corylifolia*, *Panax ginseng*, Tenmick, *Cervus Nippon,* etc. GLJC also decreased oxidative stress by reducing TBARS (thiobarbituric acid reactive substances) in the midbrain, olfactory lobe, and cortex and enhancing SOD (superoxide dismutase) activity in the hypothalamus and midbrain (64). Recently, GLJC has been evaluated for its effects and safety profile in the management of mild-to-moderate cognitive impairment in a multicenter, randomized, positively controlled double-blind clinical trial. 348 participants were given GLJC (0.6 g) once daily along with *Gingko biloba* extract (19.2 mg) thrice a day for 2 sessions over 24 weeks. The effects of GLJC were observed on cognition through MMSE, MoCA scale, Activity of daily living (ADL) score, independence, and Alzheimer’s disease Rating Scale-Cognitive Project (ADAS-Cog). Additionally, the concentration of acetylcholine and AChE in serum was measured. Safety profile was also measured through liver and kidney function tests, stool and urine tests, and vital signs of the body. If the results of the clinical trial are found positive, it may be further used as a significant information source for future interventions and the successful treatment of mild to moderate cognitive impairment (65). Recently, a 24-week, double-blind, controlled, randomized clinical trial reported the efficacy and safety profile of GLJC for the management of vascular mild cognitive impairment (VaMCI) in 96 patients (60-85 years of age). The patients randomly received GLJC or Gingko extract tablets. Cognition scores were measured using MoCA, MMSE, ADAS-Cog, and Chinese Medicine Symptom Scale (CM-SS) tests. GLJC groups showed significant improvement in MMSE, CM-SS, and MMSE scores compared to the Gingko group. The treatments also increased serum acetylcholine and decreased AChE, high-sensitivity C-reactive protein, and homocysteine levels. Hence, GLJC showed efficacy in improving cognition and the cholinergic system in VaMCI patients without any adverse effects (66).

*Huperzine-A from Huperia serrata*

Huperzine-A, a naturally occurring sesquiterpene alkaloid substance is found in the Chinese plant *H. serrata *(67). The compound penetrates the blood blood-brain barrier and inhibits AChE enzyme with high bioavailability. It has shown its role as a cognitive enhancer against neurodegeneration caused by glutamate, Aβ protein, H_2_O_2,_ and ischemia. Overall, these actions cause a reduction in oxidative stress which controls levels of apoptotic proteins and protects mitochondria (68). As a dietary supplement, Huperzine-A has also been reported to improve cognition and daily living activities in AD patients (67). A recent study has shown the effects of Huperzine-A, a natural sesquiterpene alkaloid extract from *H. serrata* on task-switching deficiency and cognitive dysfunction in AD patients in a double-blind study. The study includes 50 AD patients with mild/moderate dementia and 50 healthy subjects. The outcomes were estimated once for healthy subjects during diagnosis for determining baseline scores and twice for AD patients, first during diagnosis and later after 8 weeks of treatment. Addenbrooke’s cognitive and trial-making tests were performed for evaluation which showed significant improvements in the Huperzine-A treated group compared to their baseline performance with no adverse effect observed. Overall, it improved cognition status and was effective against task-switching defects (69).

Various phytochemicals recently investigated for the management of AD in humans are tabulated in Table 3.

***Mechanism involved in the neuroprotective role of phytochemicals in AD***

**Figure 2:**
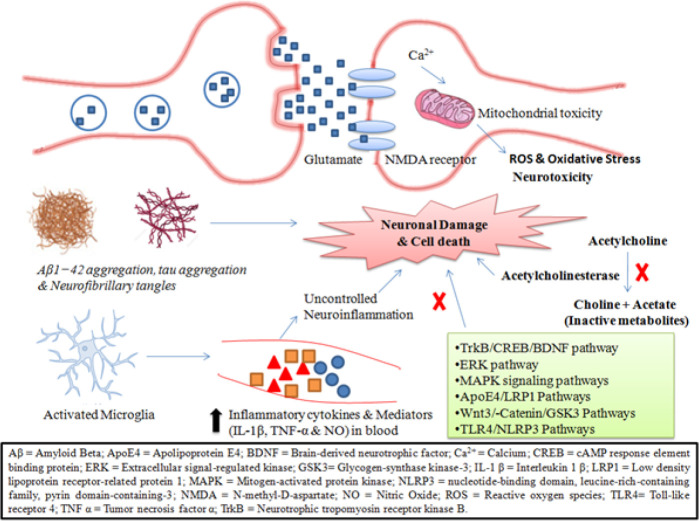
Overview of some of the mechanisms and pathways through which phytochemicals act against Alzheimer’s disease (AD)

Phytochemicals have been reported to show neuroprotective effects in AD through various mechanisms and pathways. Mostly they act as reversible AChE inhibitors to decrease the conversion of acetylcholine into inactive metabolites choline and acetate (23, 69, 70). Glutamate levels in excess are responsible for oxidative stress and ultimately neuronal death (71, 72). Attenuating ROS, oxidative stress, metabolism of APP, glutamate-induced cell death, and up-regulating antioxidant system are also responsible for their neuroprotective effects (17, 62, 65, 70, 73). Uncontrolled (17, 49, 74, 75), but also stabilized Aβ oligomers, and destabilized preformed neuroinflammation, is a key mechanism involved in the pathogenesis of AD (17). Microglial cells in the brain lead to the production of inflammatory mediators and cytokines, which ultimately leads to neuronal cell death (76). Reduction in these inflammatory mediators is one of the neuroprotective effects of phytochemicals (17, 62, 70, 75). Aβ induces memory impairment in AD models *in vivo* (77). Phytochemicals not only inhibit the formation of neurofibrillary tangles, tau phosphorylation, and Aβ aggregates fibrils but also increase the formation of microtubules (19, 78, 79). Enhancing mitochondrial membrane potential to inactivate the mitochondrial ROS scavenging system is also included among the neuroprotective mechanisms (54, 80). They also act through different pathways including TrkB/ CREB/ BDNF, ERK, MAPK, ApoE4/low-density lipoprotein receptor-related protein 1 (ApoE4/LRP1), toll-like receptor 4/ nucleotide-binding domain, leucine-rich-containing family, pyrin domain-containing-3 (TLR4/ NLRP3), and GSK3 pathways (21, 62, 73, 81, 82). Figure 2 depicts some of the most common mechanisms and pathways through which phytochemicals act against AD.

**Table 1:** Effects of phytochemicals on different Alzheimer’s disease (AD) markers investigated in *in vivo* studies

No.	Phytochemicals investigated	Animal species used	AD model used	Dose of phytochemical	Treatment schedule	Tissues/cell evaluated	Effects	Published Year	Reference
1.	Flavonoids & saponins from *Bacopa floribunda *(Native to tropoical Asia) -single herbal therapy	**BALB/c Mice**	**Aβ 1-42 induced**	**100 and 200 mg/kg**	21 days	Hippocampus	MDA, IL1β, TNF α, microgliosis decreased	2022	(24)
2	Morin, Thymol, Thymoquinone -Polyherbal combination	**Male Dawley rats**	Aluminum Chloride	Morin: 20 mg/kg Thymol: 30 mg/kg Thymoquinone: 10 mg/kg	5 weeks	Brain	Spatial recognition and memory restored; Increased HO-1 and Nrf2; Decreased TLR4 activation, Aβ and Tau hyperphosphorylation; ApoE4 and LRP1 levels back to normal	2022	(82)
3	Phytochemicals from *Curcuma longa* (native to tropical South Asia) and *Citrus Junos Tanaka *(China originated*)* -single herbal therapy	Mice	Aβ peptide-induced	-	-	Hippocampus	Reverse memory impairment; Recover cholinergic system and oxidative damage defense system, BDNF	2022	(25)
4.	Phytochemicals from *Withania somnifera*-single herbal therapy	5XFAD Mice	Aggressive amyloid deposition model	200 and 400 mg/kg/day	45 days	Frontal Cortex, entorhinal cortex, hippocampus	Ameliorated cognitive impairment, block NCX3 expression, reduced Aβ plaque, increased SOD and GSH	2022	(26)
5	Phytochemicals from naringin-single herbal therapy	Rats	Scopolamine induced	50, 100, and 200 mg/kg	14 days	Hippocampus	Effective against inhibitory passive avoidance memory deficits; increased volume, density and number of neurons in CA1 region of the hippocampus	2022	(83)
6	*Clausena harmandiana* from the Southeast Asian region) root bark extract-single herbal therapy	Male ICR mice	Scopolamine induced	100, 250, or 500 mg/kg in mice	14 days	-	Extracts (500 mg/kg) improved memory deficits	2022	(27)
7	*Forsythiae fructus* and *Cassiae semen* (traditional Chinese medicine) -Polyherbal combination	Sprague-Dawley male rats	Aβ_(25-35) _Induced	200 mg/kg	47 days	Hippocampus	Inhibition of Aβ deposition and memory deficits; Attenuation of hippocampal pAkt-pGSK-3β-pFOXO1 pathway; decrease Tau protein. No adverse effects.	2022	(84)
8	*Centella asiatica* (native to Southeast Asian)-single herbal therapy	Adult male Wistar rats	STZ	150 and 300 mg/kg	21 days	Hippocampus	Improved working memory, ameliorated neuronal loss	2022	(28)
9	Black tea extract-single herbal therapy	Female albino rats	Arsenic	1% w/v lyophilized black tea extract	28 days	Cerebellum	decreased AChE activity. No side effects.	2022	(85)
10	Jatamansinol from *Nardostachys jatamasi* (Indian Origin)-single herbal therapy	**Drosophila**	Aβ_42_	-	-	**-**	Anti-oxidant potential, inhibited cholinesterase activities, increased learning, memory and locomotor activity, decreased Aβ_42_ levels	2022	(29)
11	Mulberry Fruit Cultivar *Morus cf. nigra* ‘Chiang Mai’ (MNCM)-single herbal therapy	Drosophila (F1 progeny flies)	Short memory-deficient AD flies by co-expressing human APP and BACE-1 in CNS of fly	150–500 µg/ml	28 days	Fly brains	250 & 500 µg/ml MNCM extract reduced Aβ_1-42_, inhibited BACE-1 activity and rescued climbing index	2020	(86)
12	*Mangifera indica* -single herbal therapy	Drosophila F1 progeny flies	Co-expression of human APP and BACE-1	125 or 250 µg/ml	30 days	Brain	Decreased Aβ_1-42_ peptide formation and BACE-1 activity; amended locomotor behavior; Non-toxic profile.	2022	(30)
13	Vasicinone from *Adhatoda vasica* -single herbal therapy	Male Wistar rats	Scopolamine induced amnesia	5 mg/kg, 10 mg/kg, and 20 mg/kg	7 days	-	Improved memory and cognition in rats. No toxicity symptoms.	2023	(14)
14	*Solanum lycopersicum *(Origin – South America, Mexico, Central America) -single herbal therapy	Albino male Wistar rats	Aluminum-intoxicated rat model	500 mg/kg daily	6 weeks	-	Improvement in beam balance and T maze tests, reduced serum levels of AChE, norepinephrine, IL-6, BDNF, serotonin, MDA, and dopamine.	2024	(87)

HO-1=Heme-oxgenase-1; Nrf2=Nuclear factor erythroid 2- related factor 2; BACE-1=Beta-site APP-cleaving enzyme 1; MDA=Malondialdehyde; IL1β=Interleukin 1 beta; BDNF=Brain-derived neurotrophic factor; NCX=Na+/Ca2+ exchanger; SOD = Superoxide dismutase; GSH=Glutathione; GSK-3=Glycogen synthase kinase 3; IL6=Interleukin 6

**Table 2:** Effects of phytochemicals on different Alzheimer’s disease (AD) markers investigated in cell cultures

No.	Phytochemicals investigated	Cell type	AD model used	Phytochemical concentration	Effects	Published year	Reference
1	Betanin and Glycine betaine from *Beta vulgaris* -single herbal therapy	*In vitro* [Acetylcholine chloride as AChE substrate]	-	400 µM to 12.5 µM	Anti-AChE activity with IC_50_ values of Glycine betaine 16.41 µM and betanin 19.34 µM	2022	(32)
2	From *Rosmarinus officinalis* -single herbal therapy	*In vitro*; for cell viability-breast cancer cells (MCF-7)	-	250 µg/ml	Anti-AChE activity; IC_50_ values of extracts for anti-oxidant activity: ethyl-acetate (272 µg/ml), ethanol (387 µg/ml), aqueous (534 µg/ml).	2022	(88)
3	*Clausena harmandiana* root bark extract (native to Southeast Asian region) -single herbal therapy	*In vitro*; NG108-15 cells for H_2_O_2_ oxidative cell damage; rat C6 cells for Aβ-induced cell damage	-	Anti-oxidant=42.21 µg/ml; Anti-AChE=86.71 µg/ml; Aβ-inhibition aggregation=65.26 µg/ml; Against H_2_O_2_ oxidative cell damage and Aβ-induced cell death=100 µg/ml	Anti-oxidant activity; Anti-AChE activity; Aβ-inhibition aggregation; mitigated H_2_O_2_ oxidative cell damage; Aβ-induced cell death	2022	(27)
4	Epicatechin (EC), epigallocatechin (EGC), catechin gallate (CG), epicatechin gallate, epigallocatechin gallate (EGCG), gallocatechin gallate (GCG), theaflavin monogallate (TFMG), theaflavin digalate (TFDG) from black tea -single herbal therapy	*In vitro*	-	5 to 40 µg/ml	AChE- inhibitory activity; IC_50_ values: highest inhibition by TFDG = 1.45 + 0.06 µg/ml; TFMG= 2.38 + 0.08 µg/ml; lowest by gallic acid= 483.36 + 15.3 µg/ml. No side effects.	2022	(85)
5	Lichen species -single herbal therapy	*In vitro*	-	DPPH IC_50_ *Dactylina artica=*346.3+7.9 µg/ml;* Nephromopsis stracheyi=*595.3+64.3 µg/ml; *Tuckermannopsis Americana=*445.9+45.8 µg/ml*; Vulpicida pinastri=*283.7+31.7 µg/ml*; Asahinea scholanderi*= 1069.25+45.8 µg/ml *A. scholanderi* = 0.11±0.006 mg/ml (AChE IC_50_); 0.29±0.004 mg/ml (butyrylcholinesterase / BuChE IC_50_) *Cetraria cucullata=0.18+*0.014 mg/ml (AChE IC_50_); 0.31±0.001 mg/ml (BuChE IC_50_)	Anti-oxidant: *Dactylina artica, Nephromopsis stracheyi, Tuckermannopsis americana, Vulpicida pinastri, Asahinea scholanderi* AChE and BuChE inhibitor: alectoronic acid (strongest) & α-collatolic acid from *A. scholanderi*, usnic acid, and protolichesterinic acid from *Cetraria cucullata*	2022	(89)
6	Genistein from soybean extract (Native to Northern China) -single herbal therapy	SH-SY5Y cells	Aβ_25-35_	10, 30 or 50 µM	Cell viability increased increased HO-1 expression Nrf_2 _/HO-1/PI_3_K signaling pathway	2021	(18)
7	*Enhydra fluctuans* (flavonoids and phenolic content) – native to Southeast Asia -single herbal therapy	*In vitro*	-	AChE (IC_50_=83.90 µg/ml) and BuChE IC_50_=48.14 µg/ml) inhibition; Anti-oxidant potential (IC 50 for DPPH radical scavenging assay 113.27 µg/ml	AChE and BuChE inhibition; anti-oxidant	2021	(90)
8	Trans-chalcone Plus baicalein -polyherbal combination	*Saccharomyces cerevisiae* yeast cells	Aβ_42_	Trans-chalcone (15 µM), baicalein (8 µM)	Combination decreased ROS and Aβ_42 _levels without any effect on cell growth	2021	(91)
9	Mulberry Fruit Cultivar *Morus cf. nigra* ‘Chiang Mai’ (MNCM) -single herbal therapy	*In vitro* and *Phaeochromocytoma* PC12 neuronal cells	-	PC12 neuronal cells - 50, 100, 150, and 200 µg/ml *In vitro -* 30 mg/ml	PC12 neuronal cells -cytotoxicity, prevented H_2_O_2_ and Aβ induced toxicity, promoted neurite outgrowth. *in vitro*- Inhibited cholinesterase and beta-secretase (BACE-1) activity, anti-oxidant activity	2020	(86)
10	*Mangifera indica* -single herbal therapy	*In vitro* and Rat pheochromocytoma PC-12 neuronal cell line	-	Inhibition of AChE, BChE - 0.25 mg/ml; BACE-1- 0.125 mg/ml; Cytotoxity- 25, 50, 100 & 150 µg/ml	*In vitro* -AChE, BChE, and BACE-1 inhibition; PC12 cell- decreased H_2_O_2 _and Aβ_1-42 _induced cytotoxicity; Non-toxic profile.	2022	(30)
11	*Allium tuncelianum *(MAT) – native to Turkey -single herbal therapy	*In vitro*	-	10–30 µg/ml	Anticholinesterase activity IC_50_=11.25 µg/ml; Cytotoxic effect	2021	(92)
12	*Solanum lycopersicum*(Origin – South America, Mexico, Central America) -single herbal therapy	*In vitro*	-	0.01 and 0.05 µg/ml	AChE inhibitory (IC_50_=0.036 µg/ml) and dose-dependent anti-oxidant activity	2024	(87)
13	*Onion natrix* -single herbal therapy	*In vitro*	-	0.195 to 100 µg/ml	86% Butyrylcholinesterase inhibition compared to rivastigmine IC_50_=9 µg/ml	2024	(93)
14	*Thunbergia laurifolia* -single herbal therapy	SH-SY5Y cells	Aβ_25-35_	0-100 µg/ml (cell viability assay) 50 and 100 µg/ml for remaining assays	Cell viability- nontoxic; dose-dependent reduction in Aβ_25-35 _– induced release of lactate dehydrogenase; reduced ROS production; increased CAT and SOD activity	2024	(94)

**Table 3:** Effects of phytochemicals on different Alzheimer’s disease (AD) markers investigated in human studies

No.	Phytochemicals investigated	Participants	Type of study	Dose of phytochemical	Purpose	Duration and phase	Outcomes/ parameters	Conclusion	Published year	Reference
Healthy Individuals										
1	*Salvia officinalis* (sage), *Rosmarinus Officinalis* (Rosemary), *Melissa officinalis* (Melissa) combined extract [SRM] – Traditional European Medicine -Polyherbal combination	44 healthy subjects, 40 years and older	Randomized, placebo-controlled, double-blind pilot study	5 ml extract from 0.5 g/ml solution twice a day	To determine memory enhancement in healthy subjects by traditional European herbal medicine	2 week; Phase NA	No improvement in immediate or delayed word recall subgroup analysis significantly improved delayed word recall for subjects under 63 years of age; No serious adverse events	Effective for verbal episodic memory for 63 years of age healthy subjects	2018	(70)
Individuals at AD Risk										
2	Citrus peel extract -single herbal therapy	80 participants with subjective cognitive decline (60–75 years)	Interventional, controlled, randomized, quadruple masking	Citrus peel extracts (400 mg) standardized in auraptene 0.1 mg and naringenin 3 mg	To determine the clinical and biological effects of citrus on subjective cognitive decline	One capsule a day for 9 months; Phase NA (Pilot)	Determination of neuropsychological status and interleukin 8	Ongoing	2023	ClinicalTrials.gov Identifier (NCT number): NCT04744922
3	MIND diet for carotenoids+ Mild calorie reduction (25 kcal) - polyherbal combination	295 Community-dwelling adults, 65–84 years of age, at AD risk, overweight, having suboptimal diet	Randomized controlled intervention	MIND diet+mild calorie reduction (25 kcal)	To determine the role of the MIND diet on cognitive function.	3 years; phase 3	High Plasma α-carotene levels cause better global cognition and semantic memory scores in population at cognitive decline risk	Improved cognition by α-carotene	2021	(95)
4	*Resveratrol (present in plants of families like Gnetaceae, Vitaceae, Dipterocarpaceae, Cyperaceae, Gramineae, Poaceae, Leguminosae, Polygonaceae, Paeoniaceae)* *-* single herbal therapy	119 patients with dementia due to probable AD patients; 50 years or older	Interventional, Randomized (1:1) double-blind placebo-controlled	Starting at 500 mg once a day, increasing every 13 weeks to max of 1 gm twice daily	To determine the effect of resveratrol (SIRT-1 activator) in the treatment of mild to moderate AD patients	52 weeks; Phase 2	Safety and tolerability of drug, MRI to assess whole brain volume, AD cooperative study –activities of daily living, CSF conc of Aβ40, serious adverse events in 20.31% Res-treated participants; Non-serious adverse events – diarrhea, urinary tract infection, dizziness, and headache were observed	Limitations in study, larger study is required	2021	ClinicalTrials.gov Identifier (NCT number): NCT01504854; (96)
Severity: Mild										
5	Sesame oil cake extract (SOCE) - single herbal therapy	70 subjects with MCI; over 60 years of age	Randomized, placebo-controlled, double-blind pilot study	1.5 g/day	To determine the safety and efficacy profile of SOCE supplement on cognitive function in memory-impaired individuals.	12 week; phase NA	Decreased plasma Aβ(1-40) and Aβ(1-42) protein conc, improved verbal learning memory. Adverse events were unrelated to the test products.	Beneficial effects of SOCE in older subjects with impaired memory	2021	(81)
6	Melissa officinalis extract rich with rosmarinic acid (RA)- Native to Europe, Central Asia and Iran - single herbal therapy	23 patients with mild AD dementia	Randomized, placebo-controlled, double-blind	500 mg daily	To determine the tolerability, safety, and clinical efficacy of RA	24 week; phase NA	No serious adverse events, well tolerated, mean NPI scores improved, no improvement on disease biomarker	RA may be useful in attenuating AD-neuropsychiatric symptoms	2020	(97)
7	Sulforaphane (present in cabbage and broccoli) - single herbal therapy	160 (n=80 for Sulforaphane; n=80 for placebo; 50–75 years; mild AD patients	Interventional; randomized double-blind, placebo-controlled	2550 mg once a day	To determine the effects and safety of Sulforaphane in prodromal to mild AD patients	**24 weeks**	To be measured: AD assessment scale (cognition); daily living scores; neuropsychiatric inventory scores; MoCA scores; oxidative stress indexes and other AD-related measures	Ongoing; Not yet reported	2019	ClinicalTrials.gov identifier (NCT number): NCT04213391; (98)
Severity: Mild to Moderate										
8	VGH-AD1 (Traditional Chinese herbal medicine) - single herbal therapy	28 AD patients; mild to moderate Alzheimer’s type dementia, 65 years and older	Randomized, Interventional, placebo-controlled, double-blind, cross-over	Twice a day: Yi-gan-san 3 g, Huan-shao-dan 1 g, Danshen 0.75g, Tian-ma 0.75 g, Gou-teng 0.375 g, Ba-ji-tian 0.375g	To determine the efficacy of VGH-AD-1, a traditional herbal medicine on AD patients	8 weeks; Phase 1 & 2	To observe: GDS, MMSE, NPI-Q, IADL, IQCODE, and QoL-AD scores; Adverse effects	Ongoing study	2020	ClinicalTrials.gov identifier: NCT04249869
9	Resveratrol (present in plants of families like *Gnetaceae, Vitaceae, Dipterocarpaceae, Cyperaceae*, *Gramineae, Poaceae, Leguminosae, Polygonaceae, Paeoniaceae*) - single herbal therapy	Subset of AD subjects with CSF Aβ42<600 ng/ml	Retrospective	Starting at 500 mg once a day, increasing every 13 weeks to max of 1 gm twice daily	To observe the effect of resveratrol (SIRT-1 activator) on neuro-inflammation and adaptive immunity in AD	52 weeks	Resveratrol -*decreased metalloproteinases (MMPs) and elevated macrophage-derived chemokine (MDC), IL-4 and fibroblast growth factor (FGF)-2 in banked CSF samples. *elevated MMP10 and reduced IL-12P40, IL12P70 in plasma *improved MMSE scores, ADCS-ADL scores *stabilized CSF Aβ42; Weight Loss	SIRT-1 targeting may be useful for AD	2017, 2018	(75, 99)
10	Caffeine (present in coffee) - single herbal therapy	248 patients with AD dementia; 50 years and older	Randomized, interventional, placebo-controlled, double-blind	400 mg/day for 27 weeks, after a 3-week up-titration period	To determine the cognitive effects of caffeine in AD dementia from the starting to the moderate stage	30 weeks; Phase 3	To observe: NTB, MMSE, QoL-AD, Epworth, DAD-6, CGIC, Zarit, and NPI scores	Ongoing study	Ongoing study	ClinicalTrials.gov Identifier: NCT04570085; (100)

MIND: Mediterranean-DASH Intervention for Neurodegenerative Delay; It includes whole grains, vegetables, berries, nuts, olive oil, beans etc.; GDS: Geriatric Depression Scale; IADL: Instrumental Activities of Daily Living; IQCODE: Informant Questionnaire on Cognitive Decline in the Elderly; ADCS-ADL: Alzheimer’s Disease Cooperative Study-Activity of Daily Living; MMSE: Mini-Mental Status Examination; CGIC: Clinical Global Impression of Change; NPI-Q: Neuropsychiatric Inventory Questionnaire; DAD- Disability Assessment for Dementia; QoL-AD: Quality of Life-Alzheimer’s Disease; NTB: Neuropsychological Test battery; SIRT-1: Sirtuin-1; CSF: Cerebrospinal Fluid

High glutamate concentration leads to calcium deregulation causing calcium overload followed by depolarization of mitochondria, generation of ROS, neurotoxicity, and cell death. Aggregates of Aβ and tau along with neurofibrillary tangles are significant biomarkers of cell death in AD. Brain microglial cells also get activated which leads to excessive production of inflammatory mediators and cytokines, such as IL-1 β, TNF-α, and nitric oxide (NO) which altogether leads to uncontrolled inflammation, followed by neuronal toxicity. Acetylcholine is reduced in AD patients; Acetylcholine gets converted to choline and acetate in presence of the enzyme AChE. Phytochmeicals also play role as AChE inhibitors and act through various shown pathways. TrkB- Neurotrophic tropomyosin receptor kinase B

CREB=cAMP response element binding protein; BDNF=Brain-derived neurotrophic factor; ERK=Extracellular signal-regulated kinase; MAPK=Mitogen-activated protein kinase; ApoE4=Apolipoprotein E4; LRP1=Low density lipoprotein receptor-related protein 1; GSK3=Glycogen-synthase kinase-3; TLR4=Toll-like receptor 4; NLRP3=nucleotide-binding domain, leucine-rich-containing family, pyrin domain-containing-3

## Conclusion

AD being the most common neurodegenerative disorder follows complex pathology. Current medications are still not able to treat AD pathology fully. Derived from natural plants, phytochemicals may be included among the safest treatment options. The same is proven by extensive research and a large number of clinical trials on their usage against AD in recent years which is being carried out at present also. The current article reviewed the phytoconstituents, and measured disease markers and pathways involved in AD treatment in recent studies performed in cell cultures, *in vivo,* and in humans. The usage of various phytochemicals needs to be explored further in order to improve cognition and regeneration of lost neurons in AD.
